# Evaluating the effectiveness of continuing professional development training program: a retrospective cohort study

**DOI:** 10.1186/s12909-025-08415-w

**Published:** 2025-12-17

**Authors:** Doaa Farid, Jommel Lumibao, Fatima Safar, Lara Kassem, Zenab Gul, Shahed Al Hams, Mohamad Al Abiad

**Affiliations:** 1 CIHR Fellow in Interdisciplinary Primary Health Care Research, Family Medicine Department, School of Medicine, McGill University, Montreal, Canada; 2 Qatar Red Crescent Training, Research and Development Center, Qatar Red Crescent Society, Doha, Qatar; 3 Strategic Planning, Primary Healthcare Corporation, Doha, Qatar; 4 Business Integration Department, Aspetar Hospital, Doha, Qatar; 5 Public Relations Department, Hamad Medical Corporation, Doha, Qatar

**Keywords:** Continuing professional education, Training, Program evaluation, Knowledge acquisition, Capacity building

## Abstract

**Background:**

Continuing Professional Development (CPD) is essential for enhancing the competencies of healthcare professionals in healthcare settings. Without continuous training, quality of services and patient safety might be at risk.

**Methods:**

This quantitative retrospective cohort study analyzed paired pre- and post-test data from 538 participants across three independent CPD (Healthcare Quality and Risk Management (HQRM), Central Sterile Supply Department (CSSD), Accident Prevention for Healthcare Professionals (APHP)) delivered over 48 sessions between 2023 and 2024. Data on participant attendance, knowledge acquisition (self-assessment ratings and pre- and post-course examinations), and participant survey feedback post courses were analyzed.

**Results:**

The analysis showed that around 46.7% of participants were nurses, 36.8% allied health practitioners, 9.7% physicians, 4.7% dentists and 2.3% pharmacists, with 97.4% coming from governmental institutions. The participants’ self-perception of the courses, based on the self-assessment questionnaire, indicates a strong belief in the courses’ value and impact. All three courses resulted in highly statistically significant knowledge acquisition (*p* < .001). The mean pre-test scores improved by 38.3 to 67.1% points. Overall satisfaction was high, and participants self-reported improved competence with ratings above 96%. Key themes gathered from qualitative assessment revealed that participants plan to further professional development, implementation of new strategies in their work, and continued self-assessment to monitor progress. Future CPD topics suggested were advanced clinical skills, patient-centered care, telemedicine, and the integration of technology in healthcare.

**Conclusions:**

The courses were perceived as relevant to professional practice and associated with higher post-test knowledge scores. Future research, incorporating longitudinal follow-up is warranted to establish the definitive causal link between this training and sustained improvements in professional practice.

**Supplementary Information:**

The online version contains supplementary material available at 10.1186/s12909-025-08415-w.

## Introduction

The purpose of the continuing professional development (CPD) is to enhance healthcare professionals’ knowledge, skills, and clinical or non-clinical competence based on evidence-based best practices [1–3]. The CPD training was found to improve patient outcomes through continuous learning and empowerment of healthcare professionals [4, 5]. In some countries, such as Qatar, CPD is a regulatory requirement to maintain the quality of services for patients in the healthcare environment. However, based on the literature, CPD program attendance and access of healthcare professionals might be hindered by time constraints and lack of engagement strategies [6, 7]. Training sessions are held mostly during working hours and limited to certain language delivery, thereby deterring participation even more, which further hampers the effectiveness of such programs [1, 8]. Based on the literature review on CPD training for healthcare professionals, researchers have reported challenges in accessibility, knowledge acquisition and participation. This is due to work commitments, heavy working hours, financial constraints and language limitations [1], thereby deterring participation and hampering effectiveness of such programs [9]. Another main issue is lack of measurement of knowledge acquisition and impact; over 50% of CPD initiatives don’t track long-term results, and organizations lack easy ways of telling if their investment is paying out [4]. Ultimately, healthcare professionals are required to do continuous professional training to maintain their license and gain skills as part of continuous capacity building that would benefit patient care and health service delivery [10].

The Qatar Red Crescent Training, Research, and Development Center of Qatar Red Crescent Society (QRCS) had conducted, from 2023 to 2024, three independent CPD courses with a total of 48 sessions and a total of 538 participants. In this quantitative retrospective cohort study, secondary data analysis on (1) participant attendance, (2) knowledge acquisition and (3) participant feedback will be assessed. The study aims (1) to evaluate the participant attendance, knowledge acquisition and participant feedback from three independent CPD courses and, (2) to present recommendations on how to improve CPD course delivery aligning with healthcare professional’s needs.

## Methods

The purpose of this quantitative retrospective cohort study is to evaluate the impact of the CPD program at the QRCS using secondary data. As part of the DHP-AS CPD Activity Accreditation Standards, it states that accredited CPD activities must provide participants with the opportunity to evaluate each session and the overall CPD activity [11, 12]. The Qatar Red Crescent, as an accredited CPD Provider organization in accordance with DHP-AS Accreditation Standards for CPD Provider Organizations, had implemented a process to assess whether individual CPD activities were successful in achieving the intended outcomes. Evaluation method used include strategy or tool that promote reflection, or self-assessment by participants, measured gains in knowledge and skills, impact on competencies, attitudes, relevance to professional practice, and perceived potential for better patient outcomes. Secondary data was obtained from institutional records, including attendance logs, pre- and post-course examination scores, and post-session evaluation surveys on three different CPD training courses with 48 individual sessions delivered in 2023–2024. The courses delivered were Healthcare Quality and Risk Management (HQRM), Central Sterile Supply Department (CSSD), and Accident Prevention for Healthcare Professionals (APHP). Three outcomes (participant attendance, knowledge acquisition and feedback) will be evaluated.

The first outcome is participant attendance, which is defined as the total number of participants who physically attended a CPD session, as documented in official session logs. A descriptive analysis will be done of participants’ attendance, their affiliations (private vs. governmental), number of Credit Hours, cost, participant fees, and time frame.

The second outcome would be knowledge acquisition, defined as the improvement in clinical or professional knowledge, evaluated by conducting descriptive analysis and means/averages of individual scores from (1) pre- and post-exam scores and (2) self-assessment ratings. The pre and post-course examination questionnaires (Appendixes E, F, and G) vary in the number of items per CPD course. For self-assessment ratings, they evaluated four elements: (1) gained new knowledge, (2) impact on Competence, (3) impact on Performance and, (4) impact on Patients’ outcomes. Self-assessment used a 5-point Likert scale (ranging from 1 = Strongly Disagree to 5 = Strongly Agree), where the reported percentage reflects the proportion of participants selecting the top two options (Agree/Strongly Agree, or 4/5).

The third outcome was participant feedback, collected through a post-course evaluation questionnaire (Appendix H). All CPD courses included a combination of Likert-scale with weighted scores, yes/no, and open-ended questions for comments. The questionnaire assesses 16 evaluation domains: [1] presentation content [2], session structure [3], session pace [4], course schedule convenience [5], usefulness of course materials [6], relevance of information [7], advertising and promotional materials [8], registration process [9], venue [10], achievement of overall course objectives [11], achievement of individual session objectives [12], disclosure of conflicts of interest [13], unbiased and non-commercial presentation [14], scientifically sound, evidence-based, objective, and balanced content [15], appropriate course format, and [16] speaker performance. The evaluation questionnaire was administered in printed form at the conclusion of the course to all attendees. Participants were informed verbally that their responses and identities will be treated with strict confidentiality and anonymity, respectively.

Inclusion criteria for data analysis were attendance at the full session and completion of both the pre- and post-course examinations and the post-session evaluation surveys. Participants with incomplete paired pre- and post-test scores were excluded from the knowledge acquisition analysis. All raw scores were first converted to percentage correct scores based on the total number of items in the respective pre- and post-course exams (Appendix H, I, J)). Knowledge acquisition data were analyzed using descriptive statistics, reported as Mean and Standard Deviation (SD). To determine the statistical significance of the knowledge gain, a Paired Samples t-test was performed, comparing the mean post-test percentage score to the mean pre-test percentage score for each course. A two-tailed p-value of *p* <.05 was considered statistically significant.

## Results

### Descriptive analysis

Quantitative retrospective data analysis has been conducted on three major outcomes: participant attendance, knowledge acquisition, and participant feedback (Appendix A) Topics delivered were Healthcare Quality & Risk Management (HQRM), Central Sterile Supply Department (CSSD), and Accident Prevention for Healthcare Professionals (APHP) (Table 1). Descriptive analysis results showed that 48 sessions were delivered in 2023–2024. Around 23 sessions were done for HQRM, 16 sessions for CSSD, 9 sessions for APHP. Topics delivered were Healthcare Quality & Risk Management (HQRM), Central Sterile Supply Department (CSSD), and Accident Prevention for Healthcare Professionals (APHP). The type of CPD training was delivered through a classroom-based workshop and similar each training and courses. HQRM delivered 3.75 CPD credits of a total of 4 number of hours. CSSD training delivered 2.00 CPD credits for a total 2 number of hours. APHP delivered 7.50 CPD credits equivalent to 8 h.

**Table 1:** Description of participant attendance for CPD sessions AHP = Allied health Practitioners; HQRM = healthcare quality and risk Management; CSSD = Central sterile supply department Training; APHP = Accident prevention for healthcare professionals

CPD Course Title	Type of CPD Training Method	Course Duration (hours)	Number of sessions	Time Frame	CPD Credits	Physicians % of Attendance	Nurse % of Attendance	Others % of Attendance
HQRM	In-Person	4	23	May 11, 2023, to November 2, 2023	3.75	12%	45%	AHP: 36%, Pharmacists: 4%, Dentists: 3%
CSSD	In-Person	2	16	July 19, 2023, to October 25, 2023	2.00	6%	56%	AHP: 31%, Pharmacists: 3%, Dentists: 4%
APHP	In-Person	8	9	January 6, 2024, to March 30, 2024	7.5	11%	39%	AHP: 43%, Pharmacists: 0%, Dentists: 7%

### Participant Attendance

Descriptive analysis shows that a total of 538 participants attended the CPD seminars. In Table 2, the number of participants is summarized across three core CPD training programs organized by QRCS. Of the total, 48.14% attended the Healthcare Quality and Risk Management (HQRM) course, 46.65% attended the Central Sterile Supply Department Training (CSSD), and 5.21% attended the Accident Prevention for Healthcare Professionals (APHP) course. Based on available data, approximately 97.4% of participants were from government institutions such as the Primary Healthcare Corporation, Hamad Medical Corporation, Naufar and QRCS. Around 2.6% were from private institutions, including Al Emadi, F Medical, Al Wehda Medical Center, Rayhan Medical Complex, Atlas Medical Center and Al Esraa Polyclinic. Regarding professional roles, on average 46.67% were nurses, 36.67% allied health practitioners, 9.67% physicians, 4.66% dentists and 2.33% pharmacists.

**Table 2:** Pre- vs. Post-Test knowledge acquisition scores and statistical significance by course

Course	*N*	Pre-test Average Score, Mean (SD)	Post-test Average Score, Mean (SD)	Mean Score Improvement (percentage points)	t-value (df)	*p*-value
HQRM	259	21.99 (13.16)	89.12 (10.20)	67.13	t(259) = 76.10	< 0.001
CSSD	251	31.51 (16.48)	88.66 (11.47)	57.15	t(251) = 49.16	< 0.001
APHP	28	47.86 (17.32)	86.19 (9.30)	38.33	t(28) = 10.96	< 0.001

QRCS organized three training courses with varying financial outcomes. The first course, HQRM, had a total income of 2,700 QAR from registration fees (180 QAR per participant for 15 participants). Expenses amounted to 2,340 QAR, covering instructor rewards (1,000 QAR), admin and training staff salaries (3,500 QAR), rent and logistics (3,000 QAR), and snacks (340 QAR). Despite modest expenses, the course achieved a net income of 360 QAR. The second course, APHP, generated a higher gross income of 6,000 QAR (400 QAR per participant for 15 participants). However, expenses were also significantly higher at 4,800 QAR, due to higher instructor rewards (3,000 QAR total for 4 sessions), operations/admin (5,000 QAR), rent/utilities/logistics (5,300 QAR), and food (300 QAR). This resulted in a net income of 1,200 QAR, the highest among the three. The third course, CSSD, enrolled 20 participants at 125 QAR each, totaling 2,500 QAR income.

### Knowledge Acquisition

The three evaluated CPD courses conducted tests pre- and post-course delivery to analyze knowledge acquisition (Appendix B, includes Tables 1, 2 and 3 with HQRM: 29 items; CSSD: 38 items; APHP: 45 items). Knowledge acquisition was defined using two metrics: (1) self-assessment ratings and (2) pre- and post-exam scores.

#### Self-assessment ratings

For self-assessment ratings, they evaluated four elements using the Self-Assessment Questionnaire (Appendix I): (1) gained new knowledge, (2) impact on Competence, (3) Impact on Performance and, (4) Impact on Patients’ outcomes. The overall ratings for each were 96.81% for HQRM, 97.03% for CSSD and 97.66% for APHP.

#### Pre- and post-exam scores analysis

For pre-and post-exam scores, tests were done for each of the individual courses using the developed pre/post-course examination questionnaires (Appendix E, F and G). The average percentage correct scores per course are presented in Table 2. For the Healthcare Quality and Risk Management (HQRM) course (*N* = 259), the mean pre-test score of 21.99 (SD 13.16) increased substantially to a mean post-test score of 89.12 (SD 10.20). This 67.13%-point gain was highly significant, t (259) = 76.10, *p* <.001. Similarly, the Central Sterile Supply Department (CSSD) course (*N* = 251) demonstrated a significant knowledge increase from a mean pre-test score of 31.51 (SD 16.48) to a mean post-test score of 88.66 (SD 11.47), reflecting a 57.15%-point improvement, t(251) = 49.16, *p* <.001. Finally, the Accident Prevention for Healthcare Professionals (APHP) course (*N* = 28) showed a significant change from a mean pre-test score of 47.86 (SD 17.32) to a mean post-test score of 86.19 (SD 9.30), a 38.33%-point improvement, t (28) = 10.96, *p* <.001. In all cases, the post-test standard deviations were smaller than the pre-test standard deviations, indicating less variability and greater uniformity in knowledge level after the training (Table 2).

### Participants Feedback

#### Post-course evaluation rating results

**Fig. 1:**
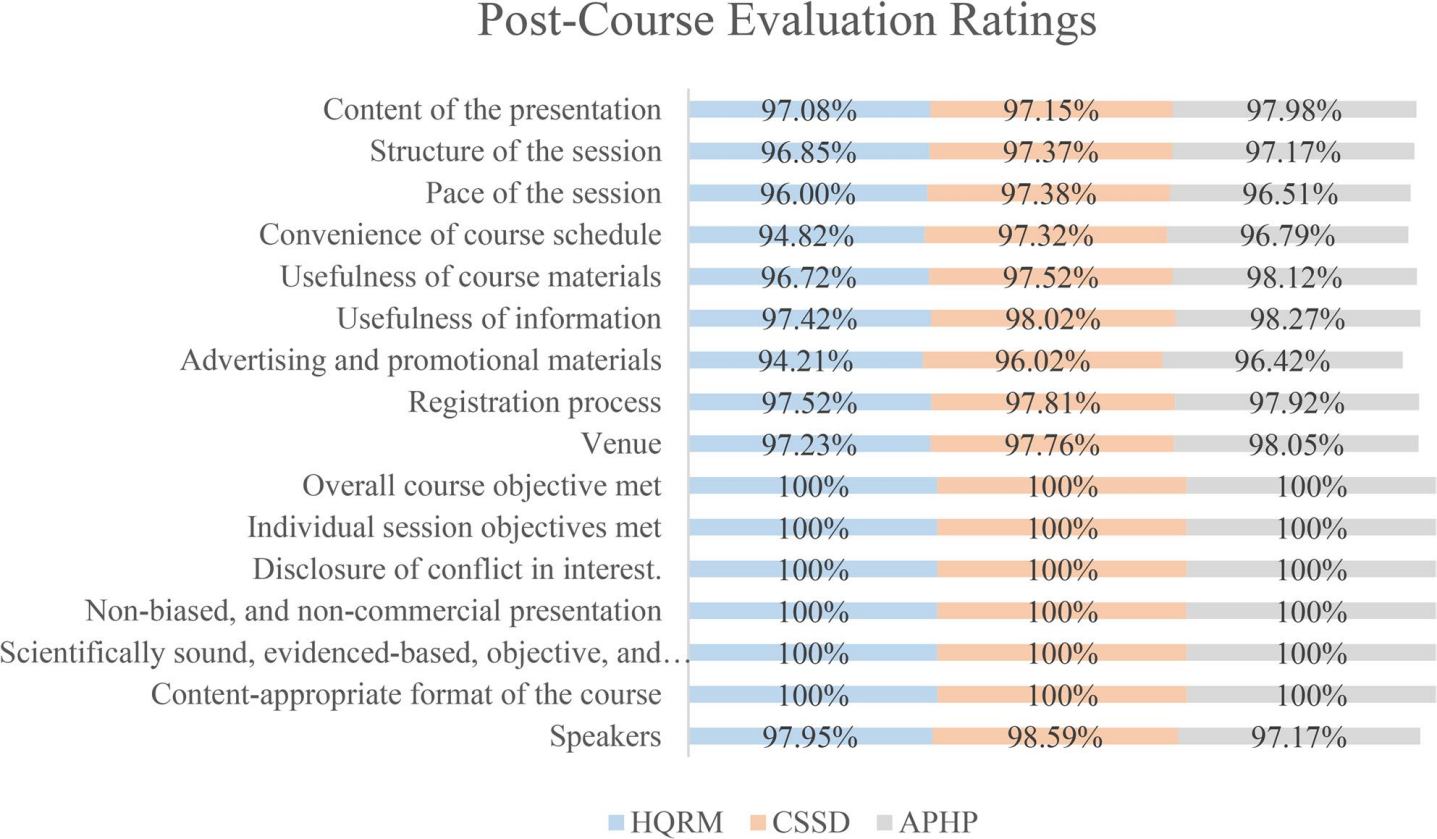
Post-Course Evaluation Ratings Results for three CPD courses. The stacked bar chart compares the 16 evaluation domains for three CPD courses: Healthcare Quality & Risk Management (HQRM), Central Sterile Supply Department (CSSD), Accident Prevention for Healthcare Professionals (APHP)

The post-course evaluation results indicate the level of satisfaction across all 16 evaluation domains, with ratings exceeding 96% in each category (Fig. 1). Evaluation domains ranged from evaluating the content of the presentation to speakers’ performance.

#### Self-reflection results

Self-reflection notes were collected post-course delivery for all CPD courses using the template for Self-Reflection Questionnaire (Appendix J). Using thematic analysis of participants’ self-reflection notes reveals several key insights such as learning satisfaction, application in practice, continuing education and practical takeaways.

**Fig. 2:**
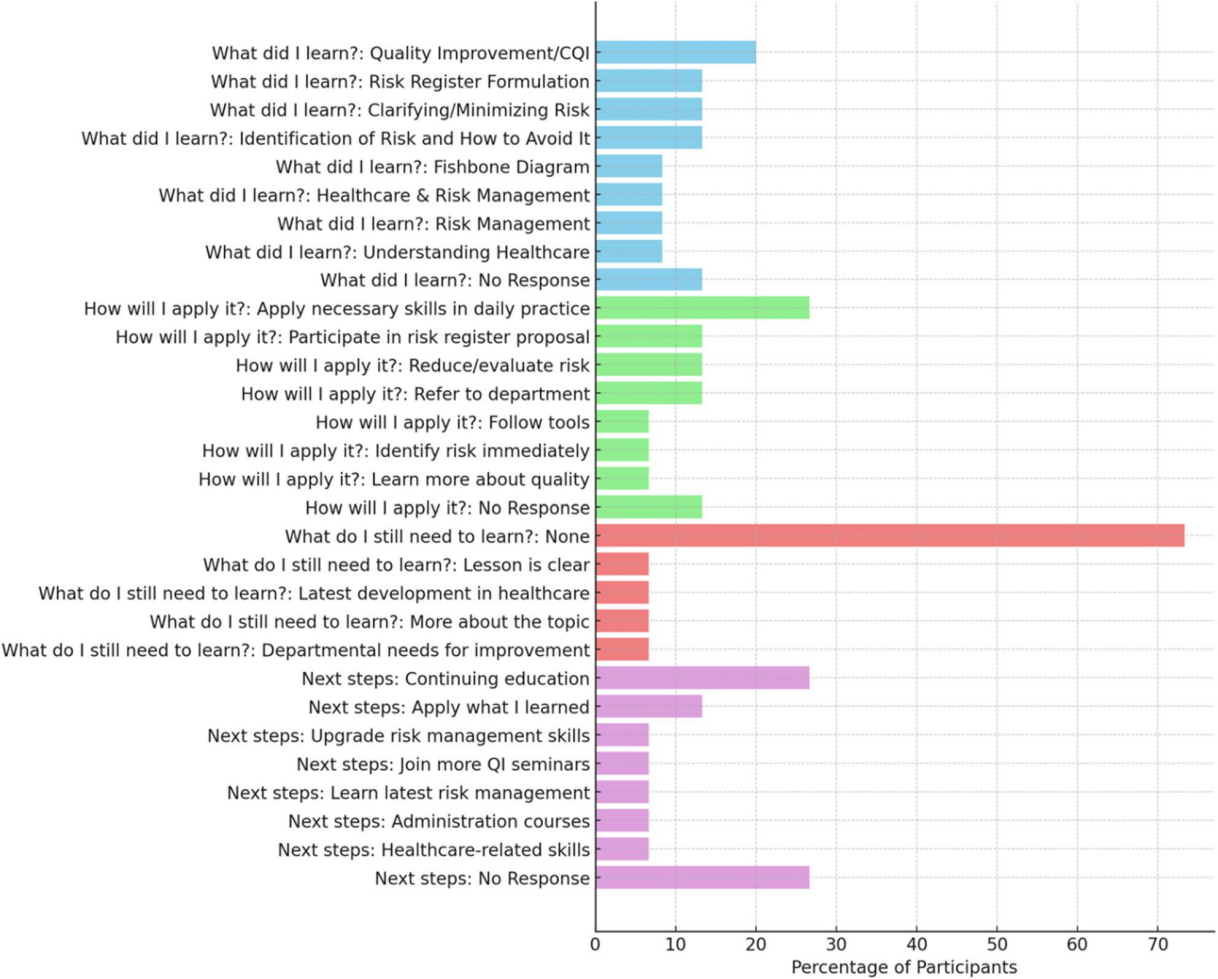
Participants Self-reflection results from three CPD courses. The stacked bar chart represents the self-reflection answers of the percentage of participants who attended three CPD courses: Healthcare Quality & Risk Management (HQRM), Central Sterile Supply Department (CSSD), Accident Prevention for Healthcare Professionals (APHP)

Participants’ self-reflection notes were collected post each course, as presented in Fig. 2. The question “What do I still need to learn?” showed that 73% of participants felt they did not need to learn anything further on the topic. In response to “How will I apply what I learned?”, 27% said they will use the knowledge in their daily practice. For the question on “Next steps,” 27% of participants mentioned a desire for continuing education. Finally, in response to “What did I learn?” 20% of participants said they learned something useful, such as how to improve quality or manage risks better (Fig. 2). Suggestions for future CPD topics included areas such as incident reporting, accreditation and credentialing, and strategic planning. To improve delivery and access, the implementation of a learning management system (LMS) is recommended to streamline participation, enable tracking, and expand the use of flexible formats, including online and hybrid training options.

## Discussion

The findings from this retrospective cohort study provide robust evidence for the immediate instructional efficacy of the three Continuing Professional Development (CPD) courses analyzed (HQRM, CSSD, and APHP). The primary result, demonstrating a highly significant knowledge acquisition across all courses (*p* <.001), suggests that the training models were effective in transferring targeted professional knowledge. The results demonstrated that the courses were perceived as relevant to professional practice and associated with higher post-test knowledge scores.

The mean post-test scores, consistently rising to above 85% correct, not only confirm efficacy but also reflect successful achievement of instructional objectives. The significantly reduced standard deviations (SDs) observed in the post-test scores indicated that the training successfully homogenized knowledge levels among participants, minimizing the initial variability and ensuring a uniform baseline of competence following course completion.

This immediate success aligns with Kirkpatrick’s first level of evaluation (Reaction) and second level (Learning), confirming the courses were positively received and led to measurable cognitive change [13]. Our results support previous literature emphasizing that well-designed, topic-specific CPD activities are crucial for maintaining and enhancing the theoretical competencies of healthcare professionals worldwide. These findings align with prior research demonstrating the positive impact of CPD on healthcare professionals’ cognitive and clinical performance [4, 5]. This perception echoes earlier studies that emphasize the role of targeted, relevant CPD in driving behavioral change and improving patient outcomes [3, 14]. The inclusion of active learning components and alignment with adult learning principles may have contributed to these outcomes [15]. The participants’ suggestions for future educational topics focused on enhancing their expertise and addressing emerging challenges in healthcare. Commonly recommended topics included advanced clinical skills, patient-centered care, telemedicine, and the integration of technology in healthcare. Participants also emphasized the importance of training in leadership, communication, and cultural competence to improve interdisciplinary collaboration and patient outcomes. Additionally, there was interest in exploring mental health, chronic disease management, and strategies for addressing healthcare disparities. These suggestions seem to reflect a desire for practical, forward-thinking education that aligns with the evolving demands of the healthcare field.

Participants’ high self-reported competence ratings (consistently above 96%) further indicate that they believe they have gained skills that are applicable to their daily roles, thereby fulfilling a critical goal of CPD. However, these self-assessment results must be interpreted with caution. As the survey instruments were developed ad hoc and were not formally validated, the high scores may reflect social desirability bias or overconfidence rather than actual, objective changes in sustained professional competence. This inherent methodological constraint suggests that self-report measures should always be supplemented with objective data. Over 70% of attendees reported no need for additional training on the same topic, suggesting that the sessions met immediate learning needs effectively. However, the fact that 26.67% still sought further training reflects a desire for longitudinal and progressively specialized CPD pathways. Several researchers recommended advocating for tiered and modular learning opportunities [1, 16].

Interestingly, governmental sector professionals represented most of the attendees. This disparity may reflect institutional and systemic barriers in the private healthcare system, including limited incentives, logistical constraints, and differing organizational cultures around professional development. Similar trends have been reported in regional and global studies, which highlight the importance of employer support, protected time, and policy alignment to enable equitable CPD participation [17–19]. The findings demonstrate the effectiveness of the QRCS CPD training program in achieving its educational and professional objectives. The multidisciplinary participation underscores the program’s relevance across various healthcare fields, fostering a collaborative learning environment. Improved post-course test scores highlight significant knowledge acquisition, validating the training’s ability to enhance participants’ understanding of key concepts. These results reflect the training’s practical relevance and alignment with professional goals. Additionally, participants’ self-reflection indicates a strong commitment to applying their learning, with many planning further professional development and suggesting future topics such as advanced clinical skills, leadership, and emerging healthcare technologies. This proactive engagement suggests the training not only meets current needs but also inspires ongoing growth.

The QRCS CPD courses demonstrated financial sustainability, generating net revenue after accounting for all expenses. This is a critical consideration for scalability, particularly as healthcare institutions worldwide face increasing financial scrutiny [20]. Leveraging cost-efficient delivery formats, such as hybrid models, could further enhance reach without compromising quality [5]. Moreover, financially viable CPD programs can be reinvested into program expansion or quality improvement initiatives, fostering a virtuous cycle of learning and institutional development.

Given increasing demands for flexibility, the integration of digital tools, such as a LMS, is recommended by other researchers [21, 22]. LMS platforms can support asynchronous, self-paced learning while ensuring content standardization, scalability, and learner tracking. This is particularly relevant in Qatar’s healthcare context, where time constraints and work shifts often impede participation in face-to-face sessions [1]. Allen and colleagues (2020) suggest blended learning models that combine face-to-face and virtual modalities [15]. These further enhance accessibility while preserving interactivity and contextual relevance and have shown promise in optimizing learning outcomes and accommodating diverse learning preferences [15, 20]. In parallel, embedding continuous feedback mechanisms, periodic needs assessments, and evidence-based instructional design will be crucial to sustaining program quality and learner engagement. Aligning CPD content with national accreditation frameworks [23] and adopting constructivist and experiential learning strategies may also enhance knowledge retention and real-world application.

## Limitations

The primary limitations of this study stem from its retrospective cohort design, which inherently limits the ability to control for all confounding variables. The study’s reliance on non-validated, ad hoc self-assessment instruments introduces the potential for response bias, necessitating cautious interpretation of the self-reported competence gains. The consistently high self-reported competence ratings (uniformly above 96%) warrant a detailed methodological note. These ratings were derived from a 5-point Likert scale (e.g., 1 = Strongly Disagree to 5 = Strongly Agree), where the reported percentage reflects the proportion of participants selecting the top two positive response options (4 or 5). This aggregate reporting method, combined with the scale’s design, likely contributes to the high percentage, potentially leading to a ceiling effect that limits the instrument’s ability to discriminate between truly excellent and merely satisfactory experiences. Furthermore, as discussed, the possibility of social desirability bias in self-assessment remains a strong influence. Therefore, while these high scores confirm strong participant satisfaction (Reaction), they must be interpreted cautiously and should be supplemented by objective performance measures in future studies. There is also a lack of long-term follow-up on knowledge retention and the objective translation of learning into practice prevents the establishment of a definitive causal relationship between the CPD courses and sustained professional performance or patient outcomes. While the knowledge gains are statistically significant, the findings may have limited generalizability beyond the local context and scope of the training programs [7, 9]. Additionally, qualitative studies exploring motivational and contextual drivers, particularly within underrepresented groups such as public sector professionals, could yield actionable insights for more inclusive and responsive CPD policies [18, 24].

## Conclusions

In conclusion, participants reported improved competence and perceived potential for better patient outcomes after the QRCS CPD training program. The value of structured CPD in supporting professional growth and improving healthcare quality emphasized the importance of aligning CPD content with learners’ professional needs and using flexible delivery formats, such as online and blended learning, to expand access and engagement. Future research needs to employ longitudinal follow-up designs post-training assessments to measure the durability of knowledge as well as integrate objective performance metrics and validated standardized instruments. The implementation of a Learning Management System could further support scalability, learner tracking, and the integration of adult learning principles. Financially, the CPD courses were sustainable, offering a model for cost-effective program expansion. Future research should assess the long-term impact of CPD on clinical practice and patient outcomes, while also exploring how organizational culture, motivation, and leadership support influence ongoing professional development. These insights will be crucial for refining CPD strategies and maximizing their contribution to healthcare workforce development.

## Supplementary Information

Supplementary Material 1.

## Data Availability

The datasets generated and/or analysed during the current study are not publicly available due internal policies but are available from the corresponding author on reasonable request.
