# Supplementary material for: Evaluating the effectiveness of continuing professional development training program: a retrospective cohort study
Source: BMC Med Educ. 2025 Dec 17;26:114. doi: 10.1186/s12909-025-08415-w (PMC12822058; doi:10.1186/s12909-025-08415-w)
Supplement: Supplementary file 1 — Supplementary Material 1. [file 12909_2025_8415_MOESM1_ESM.docx]

**Appendix A: Findings of CPD Training Program**

**Impact of CPD Training on Attendance, Feedback, and Knowledge**

The cause-and-effect diagram illustrates how the CPD training program functions as an exposure influences three key outcomes (attendance, knowledge, and participant feedback). This visual representation highlights the interconnected nature of CPD training, demonstrating its impact on attendance, participant feedback, and knowledge acquisition.

**Appendix B: Raw Data of Pre- And Post- Examination Scores for Three Courses**

| **HQRM Course** | | | | | | |
| --- | --- | --- | --- | --- | --- | --- |
| **Participant** | **Total Score Items** | **Pre-test Exam Correct Raw Score** | **Pre-test Exam Correct Percentage Equivalent** | **Post-test Exam Correct Raw Score** | **Post-test Exam Correct Percentage Equivalent** | **Improvement %** |
| Participant 1 | 29 | 4 | 13.79% | 25 | 86.21% | 72.41% |
| Participant 2 | 29 | 1 | 3.45% | 24 | 82.76% | 79.31% |
| Participant 3 | 29 | 11 | 37.93% | 28 | 96.55% | 58.62% |
| Participant 4 | 29 | 6 | 20.69% | 27 | 93.10% | 72.41% |
| Participant 5 | 29 | 6 | 20.69% | 28 | 96.55% | 75.86% |
| Participant 6 | 29 | 10 | 34.48% | 23 | 79.31% | 44.83% |
| Participant 7 | 29 | 5 | 17.24% | 25 | 86.21% | 68.97% |
| Participant 8 | 29 | 4 | 13.79% | 21 | 72.41% | 58.62% |
| Participant 9 | 29 | 13 | 44.83% | 26 | 89.66% | 44.83% |
| Participant 10 | 29 | 3 | 10.34% | 29 | 100.00% | 89.66% |
| Participant 11 | 29 | 14 | 48.28% | 29 | 100.00% | 51.72% |
| Participant 12 | 29 | 8 | 27.59% | 27 | 93.10% | 65.52% |
| Participant 13 | 29 | 5 | 17.24% | 20 | 68.97% | 51.72% |
| Participant 14 | 29 | 4 | 13.79% | 23 | 79.31% | 65.52% |
| Participant 15 | 29 | 6 | 20.69% | 28 | 96.55% | 75.86% |
|  | **AVERAGE:** | **6.67** | **22.99%** | **25.53** | **88.05%** | **65.06%** |

**Table 1: Example of *Knowledge acquisition descriptive metrics based on p*re- and *p*ost-Course Exam Scores** by participants *on one session of HQRM* Course in 2023, at Qatar Red Crescent Society.

| **CSSD Course** | | | | | | |
| --- | --- | --- | --- | --- | --- | --- |
| **Participant** | **Total Score Items** | **Pre-test Exam Correct Raw Score** | **Post-test Exam Correct Percentage Equivalent** | **Post-test Exam Correct Raw Score** | **Post-test Exam Correct Percentage Equivalent** | **Improvement %** |
| Participant 1 | 38 | 11 | 28.95% | 38 | 100.00% | 71.05% |
| Participant 2 | 38 | 8 | 21.05% | 23 | 60.53% | 39.47% |
| Participant 3 | 38 | 9 | 23.68% | 29 | 76.32% | 52.63% |
| Participant 4 | 38 | 7 | 18.42% | 28 | 73.68% | 55.26% |
| Participant 5 | 38 | 8 | 21.05% | 33 | 86.84% | 65.79% |
| Participant 6 | 38 | 8 | 21.05% | 38 | 100.00% | 78.95% |
| Participant 7 | 38 | 11 | 28.95% | 38 | 100.00% | 71.05% |
| Participant 8 | 38 | 8 | 21.05% | 32 | 84.21% | 63.16% |
| Participant 9 | 38 | 6 | 15.79% | 26 | 68.42% | 52.63% |
| Participant 10 | 38 | 6 | 15.79% | 33 | 86.84% | 71.05% |
| Participant 11 | 38 | 9 | 23.68% | 38 | 100.00% | 76.32% |
| Participant 12 | 38 | 10 | 26.32% | 34 | 89.47% | 63.16% |
| Participant 13 | 38 | 7 | 18.42% | 36 | 94.74% | 76.32% |
| Participant 14 | 38 | 7 | 18.42% | 37 | 97.37% | 78.95% |
| Participant 15 | 38 | 7 | 18.42% | 36 | 94.74% | 76.32% |
|  | **AVERAGE:** | **8.13** | **21.40%** | **33.27** | **87.54%** | **66.14%** |

**Table 2: Example of *Knowledge acquisition descriptive metrics based on p*re- and *p*ost-Course Exam Scores** by participants *on one session of CSSD* Course in 2023, at Qatar Red Crescent Society.

| **APHP Course** | | | | | | |
| --- | --- | --- | --- | --- | --- | --- |
| **Participant** | **Total Score Items** | **Pre-test Exam Correct Raw Score** | **Post-test Exam Correct Percentage Equivalent** | **Post-test Exam Correct Raw Score** | **Post-test Exam Correct Percentage Equivalent** | **Improvement %** |
| Participant 1 | 45 | 21 | 46.67% | 37 | 82.22% | 35.56% |
| Participant 2 | 45 | 27 | 60.00% | 38 | 84.44% | 24.44% |
| Participant 3 | 45 | 19 | 42.22% | 31 | 68.89% | 26.67% |
| Participant 4 | 45 | 28 | 62.22% | 41 | 91.11% | 28.89% |
| Participant 5 | 45 | 34 | 75.56% | 40 | 88.89% | 13.33% |
| Participant 6 | 45 | 34 | 75.56% | 37 | 82.22% | 6.67% |
| Participant 7 | 45 | 31 | 68.89% | 34 | 75.56% | 6.67% |
| Participant 8 | 45 | 27 | 60.00% | 36 | 80.00% | 20.00% |
| Participant 9 | 45 | 22 | 48.89% | 36 | 80.00% | 31.11% |
| Participant 10 | 45 | 29 | 64.44% | 38 | 84.44% | 20.00% |
| Participant 11 | 45 | 14 | 31.11% | 38 | 84.44% | 53.33% |
| Participant 12 | 45 | 33 | 73.33% | 36 | 80.00% | 6.67% |
| Participant 13 | 45 | 19 | 42.22% | 39 | 86.67% | 44.44% |
| Participant 14 | 45 | 12 | 26.67% | 31 | 68.89% | 42.22% |
| Participant 15 | 45 | 41 | 91.11% | 44 | 97.78% | 6.67% |
|  | **AVERAGE:** | **26.07** | **57.93%** | **37.07** | **82.37%** | **24.44%** |

**Table 3: Example of *Knowledge acquisition descriptive metrics based on p*re- and *p*ost-Course Exam Scores** by participants *on one session of APHP* Course in 2024, at Qatar Red Crescent Society.

**Appendix C:**

**Fishbone Diagram Used by QRCS Data**

**
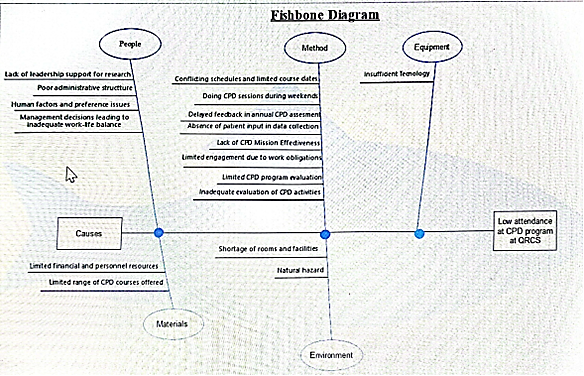
**

**Appendix E:**

**HQRM Course Pre/Post-Course Examination Questionnaire**

**
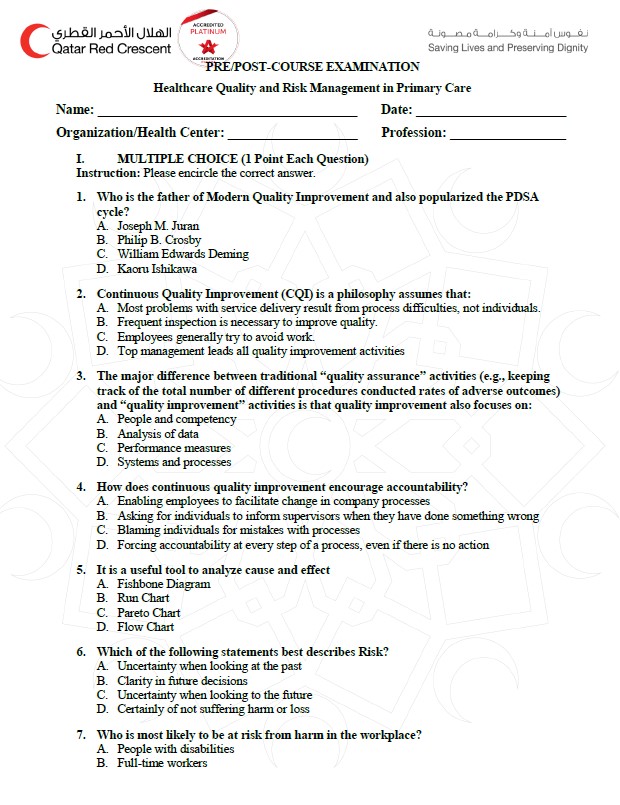
**

**
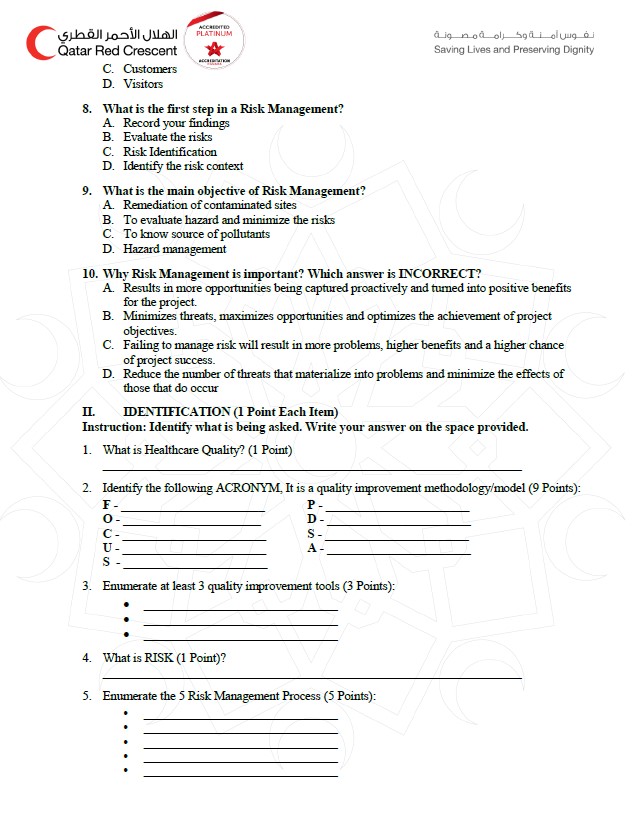
**

**Appendix F:**

**
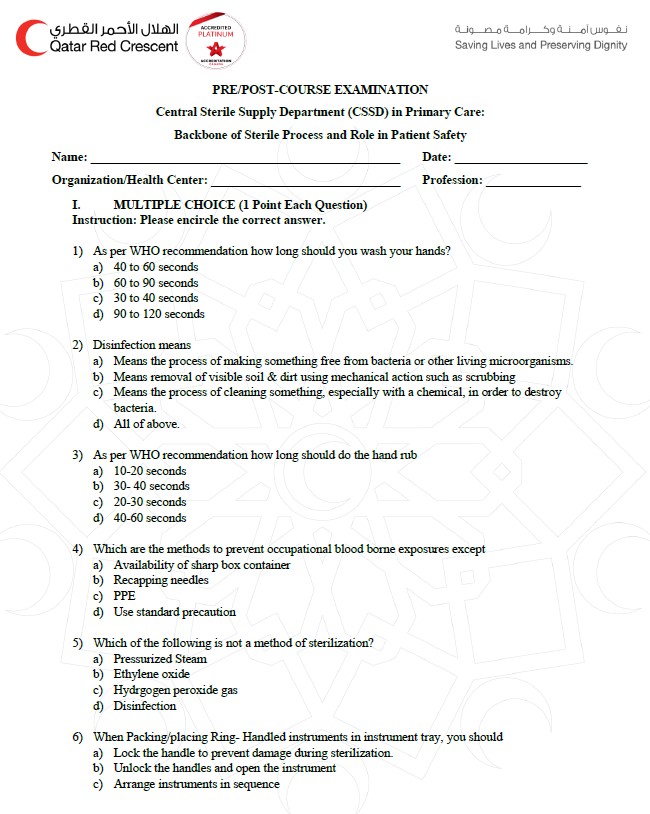
CSSD Course Pre/Post-Course Examination Questionnaire**

**
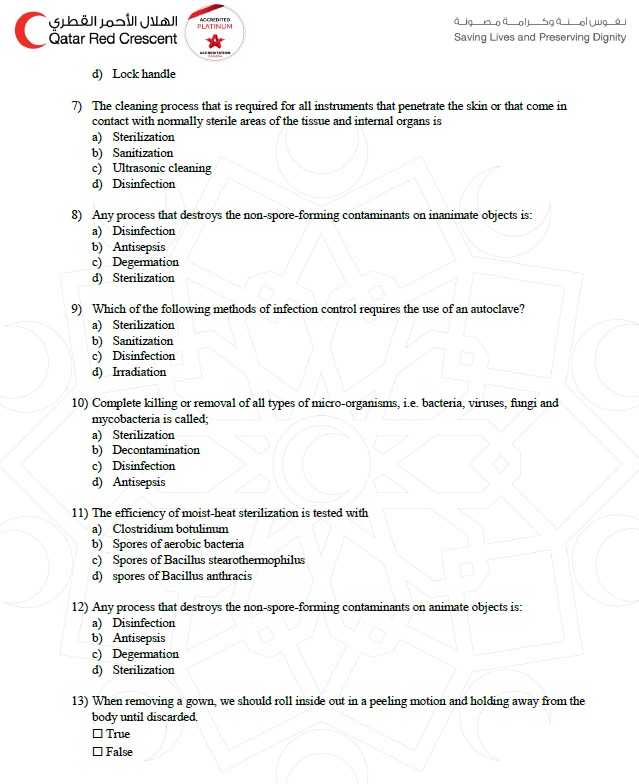
**

**
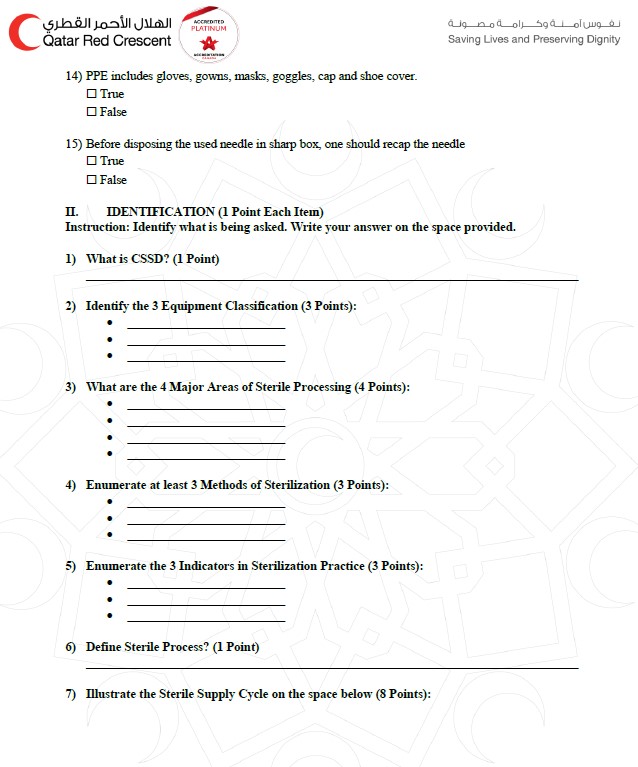
**

**Appendix G:**

**APHP Course Pre/Post-Course Examination Questionnaire**

**
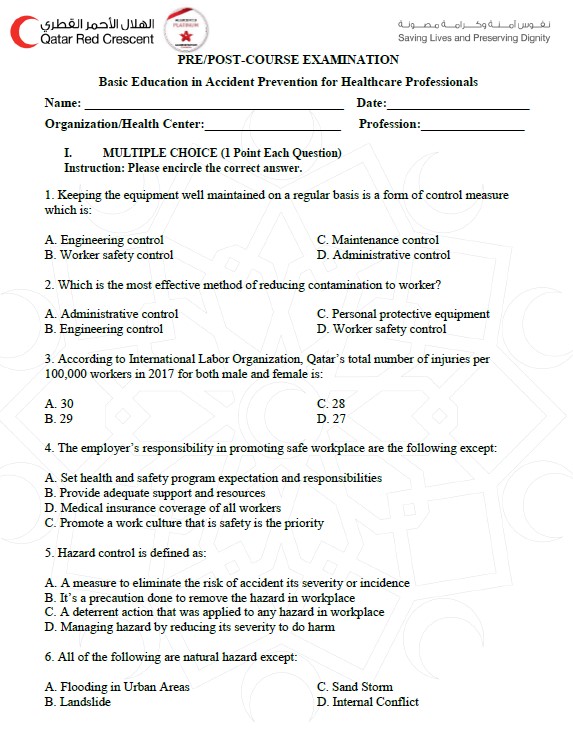
**

**
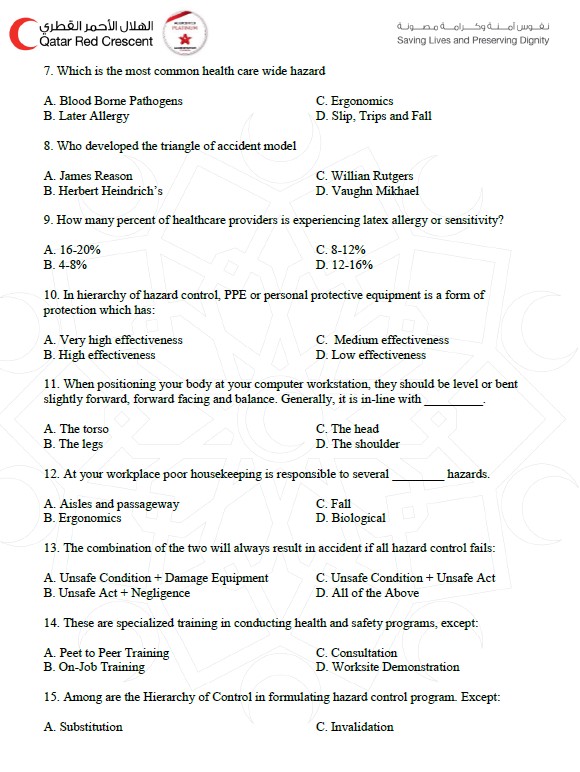
**

**
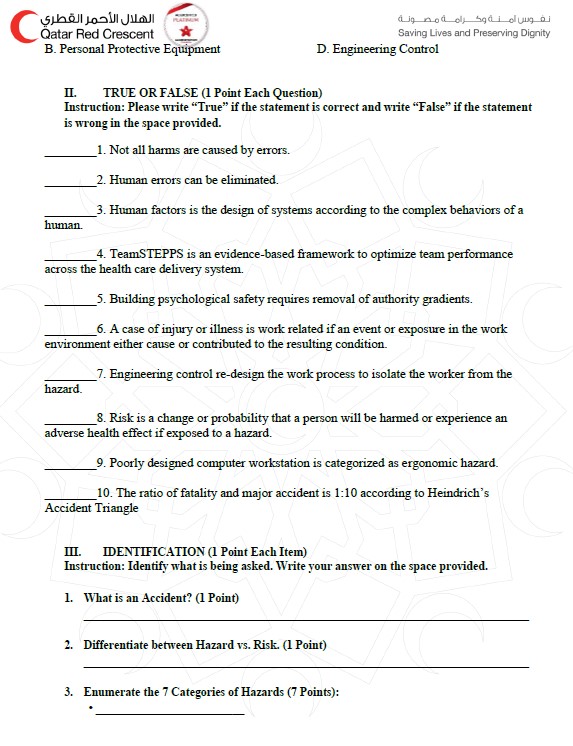
**

**
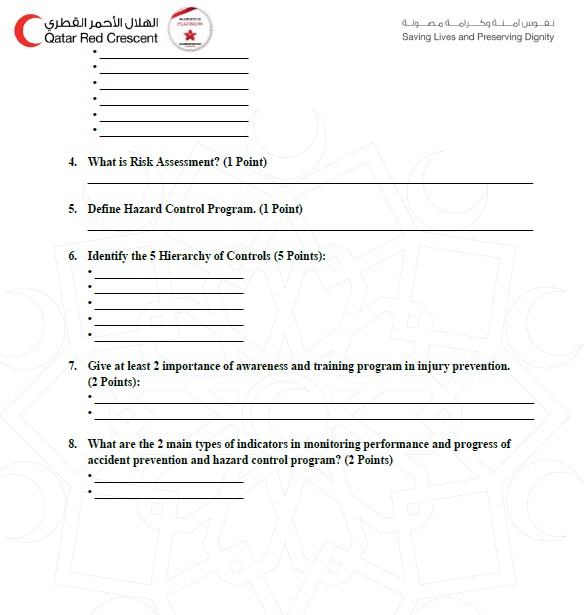
**

**Appendix H:**

**Post-Course Evaluation Questionnaire**

**
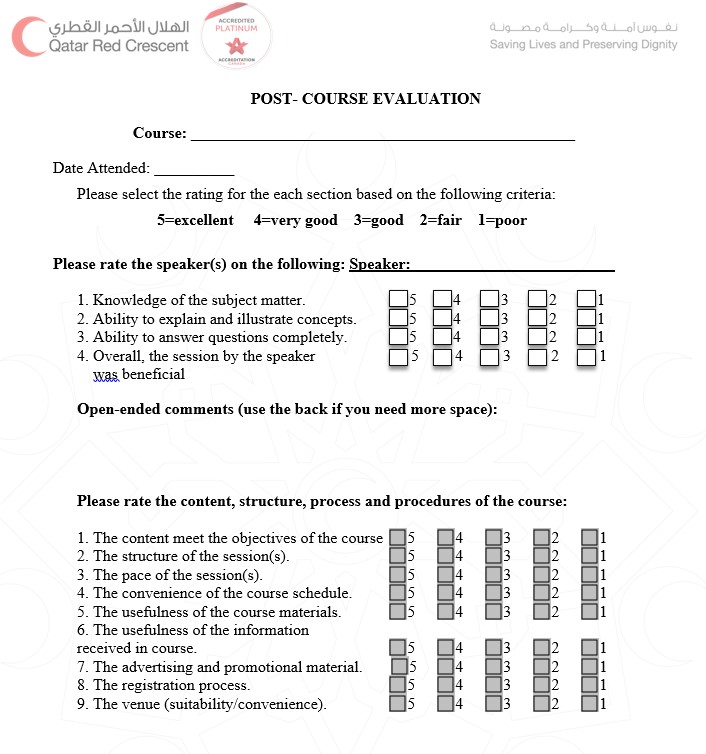
**

**
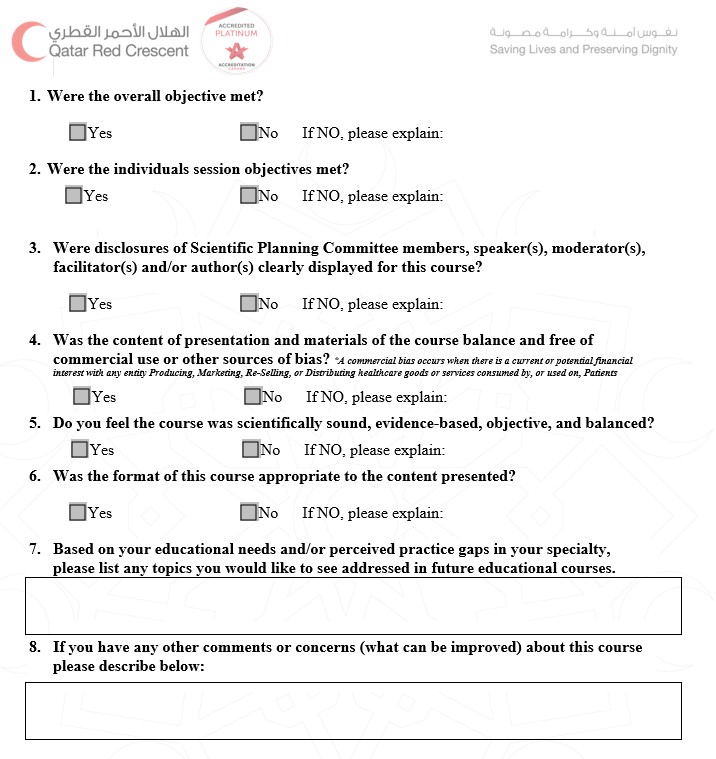
**

**Appendix I:**

**Self-Assessment Questionnaire**

**
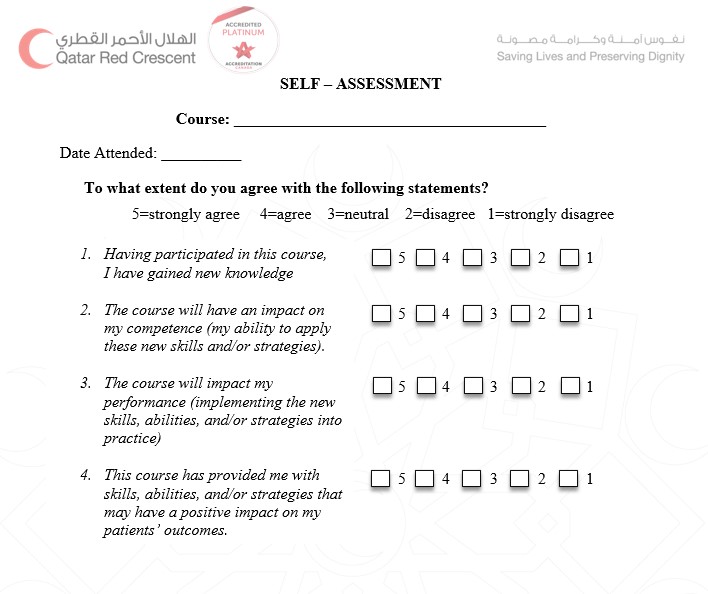
**

**Appendix J:**

**Self-Reflection Questionnaire**

**
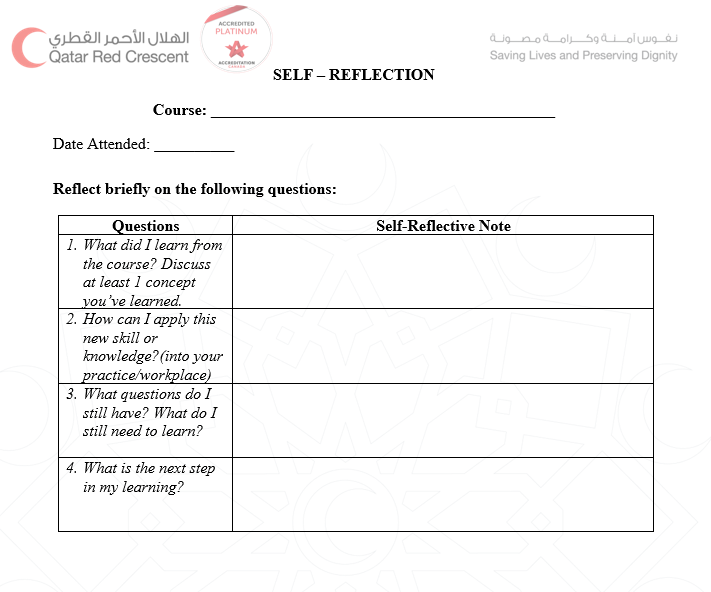
**

**
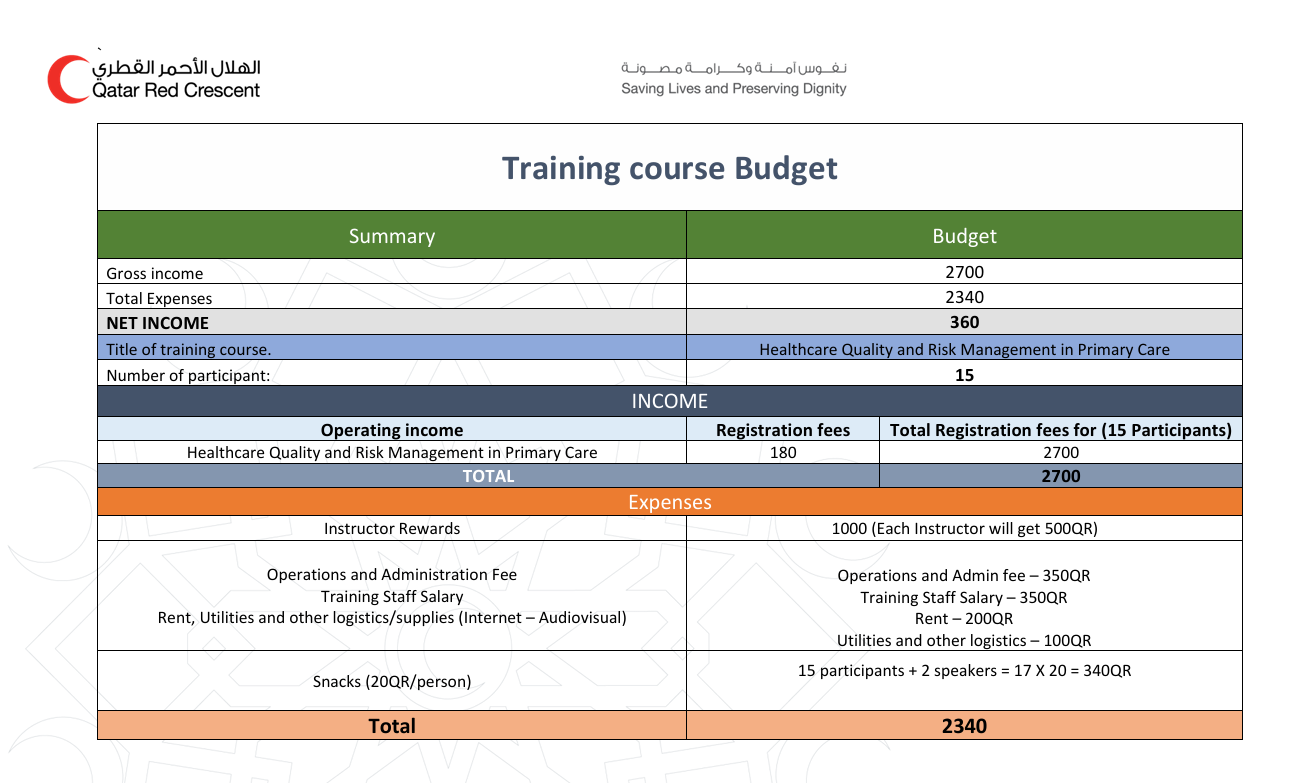
Appendix K:**

**Training Course Budget for HQRM Course**

**Appendix L:**


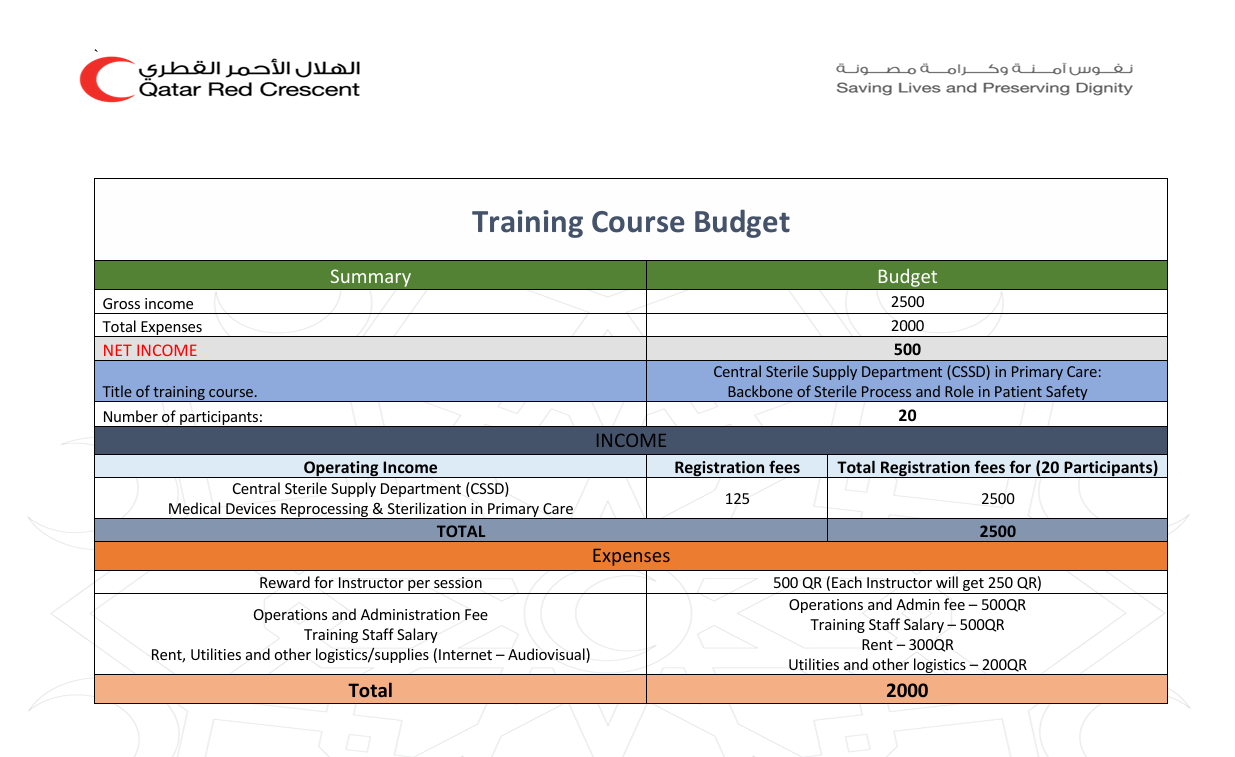
**Training Course Budget for CSSD Course**

**Appendix M:**

**Training Course Budget for APHP Course**

**
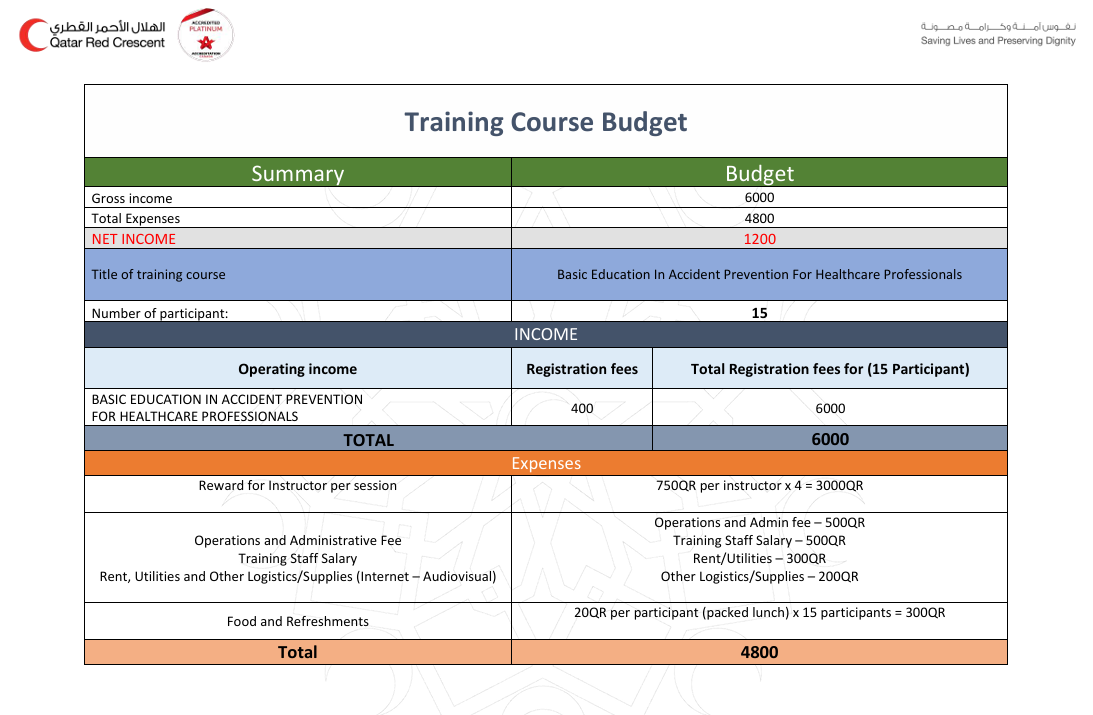
**
